# The impact of age and sex on body composition and glucose sensitivity in C57BL/6J mice

**DOI:** 10.14814/phy2.13995

**Published:** 2019-02-01

**Authors:** Thomas H. Reynolds, Allison Dalton, Lucas Calzini, Andrei Tuluca, Dakembay Hoyte, Stephen J. Ives

**Affiliations:** ^1^ Department of Health and Human Physiological Sciences Skidmore College Saratoga Springs New York

**Keywords:** Adiposity, body composition, glucose metabolism, insulin resistance

## Abstract

A paucity of data exists regarding sex differences in age‐related obesity and insulin resistance, particularly in the preclinical murine model. The purpose of this study was to determine the effects of age and sex on insulin action and body composition in C57BL/6J mice. Aged (AG, 18 months old) male C57BL/6J mice, glucose tolerance was diminished compared to young (YG, 6 months old) male mice (Area Under Curve: 95,103 ± 6818 vs. 64,005 ± 2031, *P* = 0.002). However, there was no age‐related decline in glucose or insulin tolerance in females. Body composition analysis revealed that AG males had significantly greater body mass (42.2 ± 1.9 vs. 30.0 ± 0.4 g, *P* < 0.0001), fat mass (18.7 ± 2.0 vs. 3.3 ± 0.4 g, *P* < 0.0001), body fat (43.0 ± 3.0 vs. 11.0 ± 1.5%, *P* < 0.0001) than YG males. In AG females, body mass (32.8 ± 1.6 vs. 26.3 ± 0.9 g, *P* = 0.02) was higher, but fat mass (13.3 ± 2.0 vs. 9.5 ± 1.3 g, *P* = 0.24) and body fat (37.8 ± 4.8 vs. 35.5 ± 3.8%, *P* = 0.67) were similar when compared to YG females. AG males had significantly higher body mass (42.2 ± 1.9 vs. 32.8 ± 1.6 g, *P* = 0.001) and fat mass (18.7 ± 2.0 vs. 13.3 ± 2.0 g, *P* = 0.04) compared to AG females; however, body fat (43.0 ± 3.0 vs. 37.8 ± 4.8%, *P* = 0.28) was similar. Six weeks of treatment with MitoQ, a mitochondrial‐targeted antioxidant, did not reverse age‐related obesity in male mice. Surprisingly, obesity and insulin resistance appear to be reversed in the oldest of the old male mice (28 vs. 20 months). Our findings indicate that female mice, unlike males, are protected from age‐related obesity and insulin resistance.

## Introduction

An international consortium of researchers funded by the Bill & Melinda Gates Foundation reported that nearly 30% (2.1 billion) of the world's population is overweight or obese, and this figure is projected to rise to 50% by 2030 (Ng et al. 2014). Although once considered a disease that affects only high‐income countries, such as the United States (Ogden et al. 2013), obesity is increasingly common in countries in every stage of economic development worldwide (Dinsa et al. 2012). Likewise, estimates of obesity‐related health care costs exceed $200 billion, a figure that represents 20% of US health care expenditures (Cawley and Meyerhoefer 2012). Thus, understanding the mechanisms, and possible treatments, of obesity is paramount in reducing healthcare costs and improving quality of life.

To further our knowledge regarding the mechanism that regulate obesity, hundreds of studies have used high fat feeding regiments in wild type and genetically altered mice. Such studies have greatly enhanced our understanding of diet‐induced obesity and factors contributing to the regulation of body mass. However, lagging far behind the abundant preclinical investigations regarding diet‐induced obesity is the potential impact of aging. This is concerning as it appears that there is a greater prevalence of obesity in middle‐aged men and women (40–59 years) and in older women (≥60 years) compared to their younger counterparts (20–39 years) (Ogden et al. 2014). Obesity is highly associated with insulin resistance and type 2 diabetes. Since the prevalence of obesity appears to be higher in older individuals, it would be expected that the prevalence of insulin resistance and type 2 diabetes would also be higher. Indeed, Karakelides et al. (2010) demonstrated that age‐related declines in insulin sensitivity were likely due to increases in adiposity rather than a direct age effect, further implicating obesity as a primary cause of insulin resistance and perhaps type 2 diabetes.

Obesity is associated with higher levels of reactive oxygen species and oxidative stress (Vincent and Taylor 2006) which is thought to induce insulin resistance (Houstis et al. 2006), leading to the idea that anti‐oxidants might prevent or reduce the insulin resistance associated with obesity (Abdali et al. 2015). Interestingly, treating mice with ascorbic acid (vitamin C) or MitoQ, a mitochondrially targeted antioxidant, prevents the development of diet‐induced obesity and insulin resistance (Fink et al. 2014; Lee et al. 2018). Similar to obesity and insulin resistance, aging is also associated with increased oxidative stress, although whether elevated reactive oxygen species shorten or extend lifespan has been questioned recently (Liochev 2013).

There is little information regarding the effect of aging on obesity and insulin action in the murine model (Leiter et al. 1988; Gregg et al. 2016; Krishna et al. 2016), despite this, species widespread use as a preclinical model of metabolic disease. Further, to the best of our knowledge, no information exists regarding the effect of sex on age‐related obesity and insulin resistance in mice. Therefore, the purpose of this study was to determine if sex plays a role in the development of age‐related obesity and insulin resistance. This was accomplished by assessing body composition and insulin action in male and female C57BL/6J mice at six and 18 months of age. C57Bl/6J mice are the most common inbred strain used for metabolic experiments and are highly susceptible to diet‐induced obesity and insulin resistance (The Jackson Laboratory, www.jax.org/strain/000664). Because aging and obesity are both associated with oxidative stress, we also determined if MitoQ, a mitochondrially targeted antioxidant, could attenuate age‐related obesity and insulin resistance. Our results indicate that female mice are protected from age‐related obesity and insulin resistance, and that unlike diet‐induced obesity, MitoQ does not reduce adiposity or improve insulin action in AG mice. Finally, although male mice are obese and insulin resistant at 18 months of age, body composition and glucose tolerance improve dramatically by 28 months of age.

## Methods

### Animals

**Figure 1:**
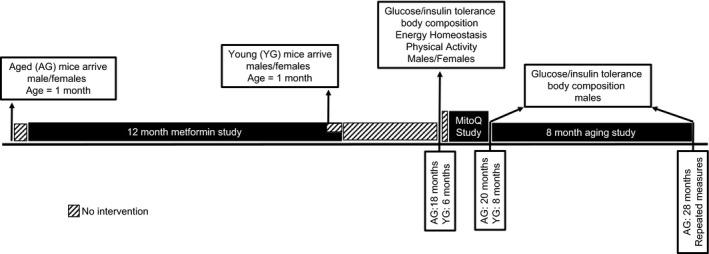
A schematic overview of the experimental design.

C57BL/6J were purchased from Jackson Laboratories (Bar Harbor, ME) at 4 weeks old and were allowed to age in the animal facility at Skidmore College. Mice were housed singularly with cage enrichment nestlets. When mice were six (YG) or 18 (AG) months old, body composition, energy expenditure, and insulin action were assessed (see below). Prior to this study, AG mice participated in a year‐long metformin study from the age of 2 to 14 months. However, mice supplemented with metformin in drinking water (0.05 mg/mL) exhibited no changes in body composition or glucose tolerance, and the mice were allowed to recover for 4 months prior to collecting data for this study. Mice were fed a standard rodent diet ad libitum (Purina RMH 3000) unless noted otherwise. An overview of our experimental design is shown in Figure 1. At the completion of the study mice were euthanized by cervical dislocation (females at 18 months, males at 28 months) while unconscious due to an intraperitoneal injection of a 1:1:1 mixture of promace, ketamine hydrochloride, and xylazine (0.015 mL/10 g body weight). All experimental protocols here were approved by the Skidmore College's Intuitional Animal Care and Use Committee (IACUC).

### Glucose and insulin tolerance testing

For glucose tolerance testing mice received an intraperitoneal injection of glucose (2.0 g/kg body weight) following an overnight fast. Tail vein blood was collected (3–5 *μ*L) at 0, 20, 40, 60, and 90 min following the injection and blood glucose was measured using a hand‐held glucometer (Accu‐Check, Roche Diabetes Care, Inc). Because it is difficult to obtain sufficient blood volume during a GTT, to supplement the GTT and parse out the potential for mice to maintain their glucose tolerance by augmenting insulin secretion, we also conducted insulin tolerance testing. Mice were allowed to recover for 7–10 days before assessing insulin tolerance. For insulin tolerance tests, an identical procedure was performed except mice were injected with insulin (0.75 U/kg body weight) following a 6‐h fast.

### Body composition

Body composition was assessed in mice by an LF50‐BCA Minispec (Bruker, Inc). The Minispec is a nuclear magnetic resonance (NMR) system that allows for the quantification of fat mass, lean mass, and free body fluid. NMR scans were completed by immobilizing fully conscious mice in a plastic tube that was placed in the instrument's sample chamber for approximately 90 sec. Body composition in mice assessed by NMR is highly correlated to the chemical analysis method of body composition assessment (Halldorsdottir et al. 2009).

### Energy expenditure and physical activity

To assess energy expenditure and physical activity, mice were single‐housed in Oxymax system metabolic cages equipped with an infrared motion detection system (Columbus Instruments, Columbus, OH). After 8 h to allow for acclimation to the metabolic cages, O_2_ consumption and CO_2_ production were measured for 24 h. During this time mice had free access to water and food. All measurements were collected at room temperature (21 ± 1°C). Energy expenditure during the light and dark cycles were calculated using the calorific value (3.815 + 1.232*RER)*VO_2_ and expressed as kcals/day. Total physical activity was assessed by counting infrared beam breaks in the horizontal (lateral movement) and vertical (rearing, jumping) planes.

### Caloric intake

Caloric intake was assessed by giving mice a known amount of chow for a given period of time (3–10 days). Remaining chow was weighed and subtracted from the initial amount of chow, multiplied by the kcal/g for each diet, and expressed as kcal/day.

### MitoQ treatment of AG male mice

MitoQ was dissolved in drinking water of AG male mice (MitoQ‐Mitochondrial Science, Auckland, New Zealand). Mice consumed MitoQ supplemented water for a total of 6 weeks. Since the initial dose (250 *μ*mol/L) reduced water intake, mice consumed water supplemented with 125 *μ*mol/L MitoQ from 2 weeks. The MitoQ dose was then we increased to 187.5 *μ*mol/L for the remainder of the study (4 weeks).

### Data and statistical analysis

Data were analyzed using Statview statistical software (SAS, Cary, NC). Data were analyzed using one way or two way (age × sex) analysis of variance (ANOVA), as applicable. Significance was established at *P* < 0.05. Data are presented as means ± SD.

## Results

### Effects of age and sex on in vivo insulin action

**Figure 2:**
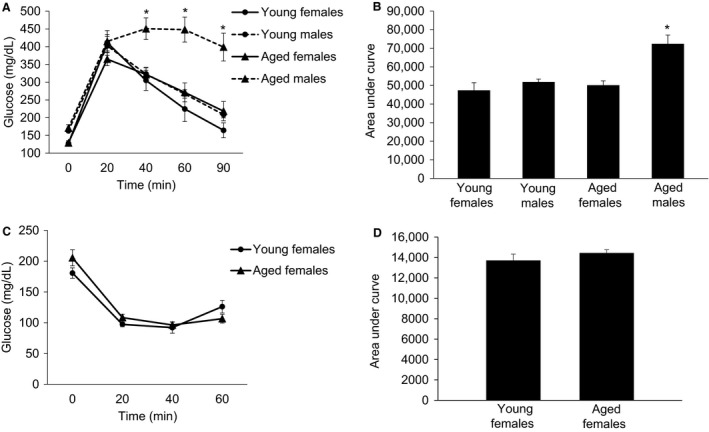
The effect of age and sex on glucose tolerance and insulin tolerance in C57BL/6J mice. (A) shows the blood glucose response to an intraperitoneal injection of glucose (2 g/kg). (B) is the area under the glucose tolerance curve. (C) shows the blood glucose response to an intraperitoneal injection of insulin (0.75 U/kg). (D) is the area under the insulin tolerance curve. Following a significant 2 × 2 ANOVA the LSD post‐hoc test was used to locate statistically significant differences. *Significantly different from all other groups by LSD post‐hoc test. *N* = 6–9 mice per group.

Despite the prevalence of insulin resistance in older individuals, there is little data regarding how sex and age interact with respect to insulin action. Therefore, we assessed in vivo insulin action in YG female, AG female, YG male, and AG male C57BL/6J mice. As shown in Figure 2A and B, aged female mice do not experience an age‐related decline in glucose tolerance; however, male mice have a significant deterioration in glucose tolerance between the ages of 6 and 18 months. Again, due to difficulties in obtaining sufficient blood volume during a GTT to measure insulin, we also conducted insulin tolerance tests in YG and AG female mice and, importantly, demonstrated that aging did not impact insulin tolerance (Fig. 2C and D).Our findings indicate that female mice are protected from age‐related insulin resistance.

### Effects of age and sex on body composition

Although advancing age is associated with obesity, there is little data regarding how sex impacts age‐related obesity. Therefore, we assessed body composition using nuclear magnetic resonance spectroscopy (NMR). We demonstrate that aging results in significant weight gain in both male and female C57BL/6J mice; however, the relative increase in body mass in male mice appears to greatly exceed that of female mice (Table 1). The age‐related increase in body mass, at least in males, is predominantly due to an increase in adiposity, as fat mass increased by 6‐fold. Despite body mass being higher in AG compared to YG female mice, fat mass was not statistically significant, increasing only 1.4‐fold. Aligned with the fat mass data, we show that AG male mice experienced a significant 4‐fold increase in % body fat with advancing age, whereas in female % body fat did not change significantly with advancing age. As expected, lean mass decreased significantly in AG males compared to YG males demonstrating age‐related sarcopenia. Interestingly, lean mass actually increased with aging in female mice. Finally, we demonstrate that free body fluid, a component of body composition derived from NMR, significantly increased with advancing age in both male and female mice, perhaps indicating inflammation or end organ dysfunction (heart/renal failure). These findings indicate that female mice are less susceptible to age‐related obesity than male mice.

**Table 1:** The effects of age and sex on body composition of C57BL/6J mice

	Young female(*n* = 8)	Aged females(*n* = 11)	Young males(*n* = 8)	Aged males(*n* = 17)	ANOVAAge effect*P*‐value	ANOVASex effect*P*‐value	ANOVAAge × Sex*P*‐value
Body mass (g)	26.34 ± 0.92	32.81 ± 1.61*	29.34 ± 0.95	42.16 ± 1.85*, +	0.0001	0.0008	0.1156
Fat mass (g)	9.55 ± 1.33	13.25 ± 2.09	3.27 ± 0.43^T^	18.67 ± 2.00*, +	0.0001	0.8387	0.0080
Lean mass (g)	14.67 ± 0.55	16.86 ± 0.57*	22.64 ± 0.54#	18.84 ± 0.40*, +	0.1371	0.0001	0.0001
% Body fat	35.46 ± 3.81	37.83 ± 4.76	10.96 ± 1.48#	42.96 ± 3.01*	0.0001	0.0152	0.0004
Free body fluid (g)	2.41 ± 0.12	2.97 ± 0.15*	2. 18 ± 0.01	3.23 ± 0.14*	0.0001	0.933	0.1198

* Denotes a significant difference from young mice of the same sex, *P* ≤ 0.05.

+ Denotes a significant difference from aged female mice, *P* ≤ 0.05.

# Denotes a significant difference from young female mice, *P* ≤ 0.05.

T Denotes a trend from young male mice compared to young female mice, *P* = 0.064.

### Effects of age and sex on energy homeostasis

**Figure 3:**
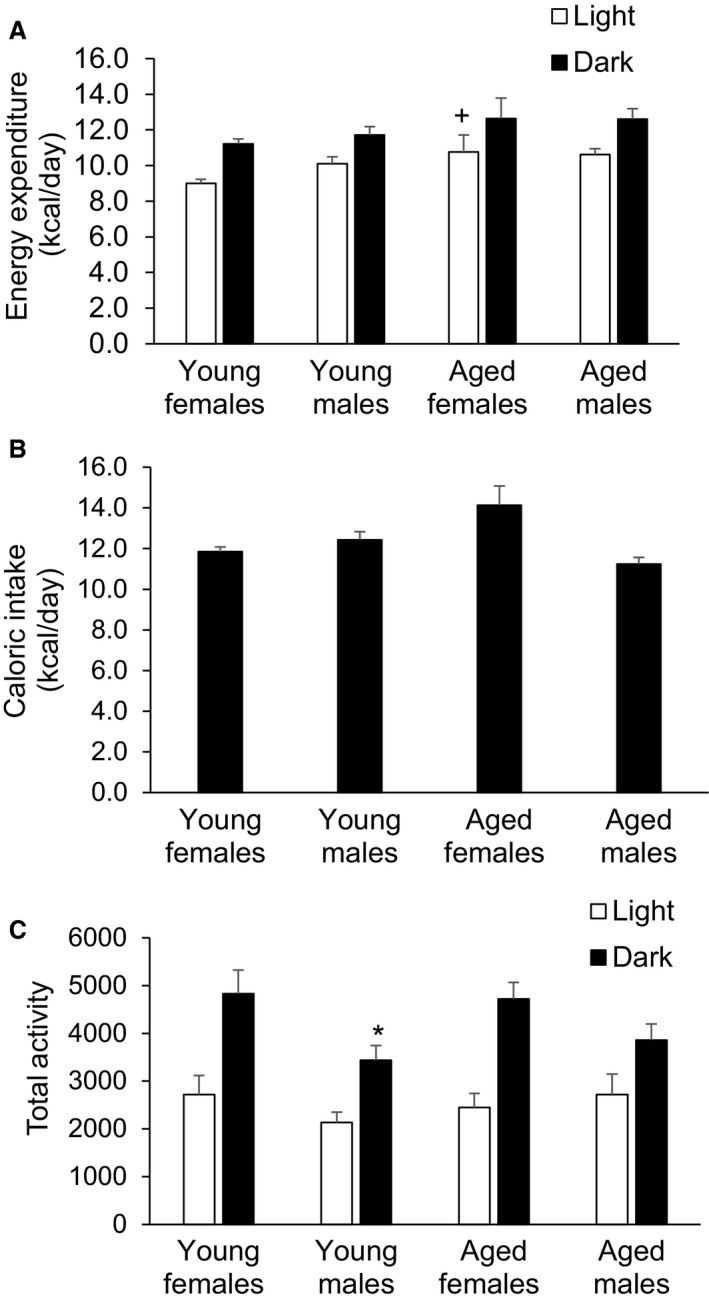
The effect of age and sex on energy expenditure (A), caloric intake (B), and physical activity (C) in C57BL/6J mice. Following a significant 2 × 2 ANOVA, the LSD post hoc test was used to locate statistically significant differences. ^+^Significantly different from young females during the light cycle by LSD post‐hoc test. *Significantly different from young females in the dark cycle by LSD post‐hoc test. *N* = 8 mice per group.

Because we observed such profound effects of sex and age on body composition in C57BL/6J mice, we determined whether or not energy expenditure, caloric intake, and physical activity played a role. A 2 × 2 ANOVA revealed a significant main effect for age (*P* = 0.047) for energy expenditure during light period, while during the dark cycle there was only a trend for an age main effect (*P* = 0.106), with no sex main effect or age × sex interaction during either the light or dark periods (Fig. 3A). Post hoc testing revealed that the age related differences in energy expenditure during the light period were due to the AG female mice being higher than the YG female mice (*P* = 0.031). There were no significant age‐ or sex‐related difference in calories consumed per day (Fig. 3B).With regard to total activity, a 2 × 2 ANOVA revealed a significant main effect for sex (*P* = 0.006) during the dark period with no significant effect during the light period (Fig. 3C). Post‐hoc testing revealed that the sex related differences in total activity during the dark period were due to the YG female mice being higher than the YG male mice (*P* = 0.015). Although total activity in the dark period was substantially higher in AG female mice compared to AG male mice, this difference was not statistically significant (*P* = 0.12). To summarize, it does not appear that differences in energy homeostasis explain the large differences in body composition observed between AG male and female mice.

### Effects of MitoQ treatment on insulin action and body composition

Because aging is associated with increased oxidative stress (Liochev 2013), we decided to treat our AG male mice with MitoQ, a potent mitochondrial‐targeted antioxidant that has been shown to improve insulin action and reduce diet‐induced obesity (Fink et al. 2014). Initially, we treated mice with 250 *μ*mol/L of MitoQ dissolved in drinking water; however, this dose greatly reduced water intake so we decreased the dose to 125 *μ*mol/L. Following 2 weeks of treatment with 125 *μ*mol/L MitoQ, we then increased the dose to 187.5 *μ*mol/L for an additional 4 weeks and observed no decreases in water consumption.

**Figure 4:**
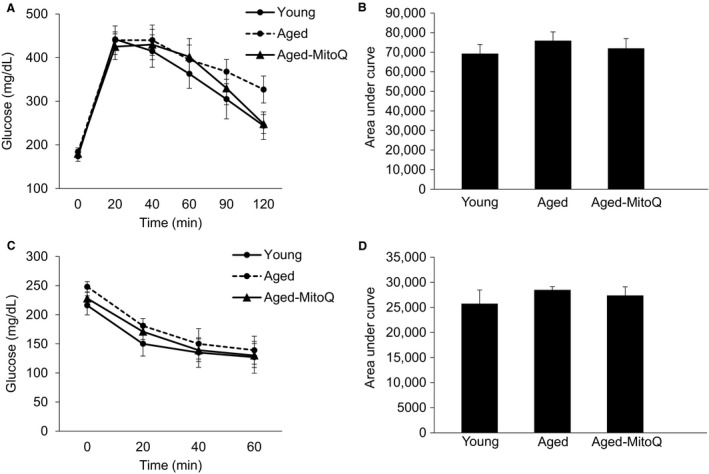
The effect of 6 weeks of MitoQ treatment on glucose tolerance and insulin tolerance in aged C57BL/6J mice. (A) shows the blood glucose response to an intraperitoneal injection of glucose (2 g/kg). (B) is the area under the glucose tolerance curve. (C) shows the blood glucose response to an intraperitoneal injection of insulin (0.75 U/kg). (D) is the area under the insulin tolerance curve. *N* = 6–7 mice per group.

Six weeks of MitoQ treatment did not appear to have any significant effect on glucose tolerance (Fig. 4A and B) or insulin tolerance (Fig. 4C and D). Surprisingly, the severe insulin resistance that was observed in AG male mice prior to the initiation of MitoQ treatment was abolished, indicating either an improvement in insulin action in the AG mice or a deterioration of insulin action in the YG mice. In other words, the severe age‐related insulin resistance that was observed in male mice at 18 months was no longer present at 20 months of age. This serendipitous finding suggests that from the age 18 to 20 months age‐related insulin resistance is reversed.

With regard to the effect of MitoQ treatment on body composition, a 2 × 3 ANOVA with repeated measures on treatment (pre‐ vs. posttreatment) revealed a significant main effect for group (YG control vs. AG control vs. aged MitoQ, *P* ≤ 0.05), a significant time effect (pre‐ vs. posttreatment, *P* ≤ 0.05), and a significant time × group interaction (*P* ≤ 0.05) for all body composition components (Table 2). We then conducted LSD post‐hoc test to locate statistically significant differences. Although the YG control mice were significantly different from both the AG control and AG MitoQ mice, no differences existed between the AG control mice and the AG mice that were treated with MitoQ. These findings indicate that age‐related obesity, unlike diet‐induced obesity, is not reduced with MitoQ treatment.

**Table 2:** Six weeks of MitoQ treatment does not alter body composition of aged C57BL/6J male mice

	Pretreatment			Posttreatment		
	Young control (*n* = 8)	Aged control (*n* = 7)	Aged MitoQ (*n* = 8)	Young control (*n* = 8)	Aged control (*n* = 7)	Aged MitoQ (*n* = 8)
Body mass (g)	32.55 ± 0.89	43.16 ± 2.95*	42.00 ± 2.44*	32.04 ± 0.77	39.59 ± 2.45*	38.98 ± 2.57*
Fat mass (g)	5.33 ± 0.70	20.14 ± 3.23*	18.07 ± 2.50*	6.29 ± 0.93	15.84 ± 2.69*	14.03 ± 2.81*
Lean mass (g)	22.11 ± 0.58	18.17 ± 0.58*	19.31 ± 0.49*	21.76 ± 0.34	19.25 ± 0.44*	20.27 ± 0.57*
% Body fat	16.54 ± 1.93	45.16 ± 3.91*	41.74 ± 3.30*	18.98 ± 2.21	38.48 ± 4.23*	34.02 ± 4.40*
Free body fluid (g)	2.29 ± 0.07	3.11 ± 0.24*	3.90 ± 0.19*	2.63 ± 0.07	3.32 ± 0.29*	3.08 ± 0.21

2 × 3 ANOVA with repeated measure on treatment (pre vs. posttreatment) demonstrated significant main effects for group and treatment and a significant group × treatment interaction for all variables (*P* ≤ 0.05).

* Denotes a significant difference from young control mice at same time point by post hoc LSD (*P* ≤ 0.05).

### Advancing age improves glucose tolerance and body composition

**Figure 5:**
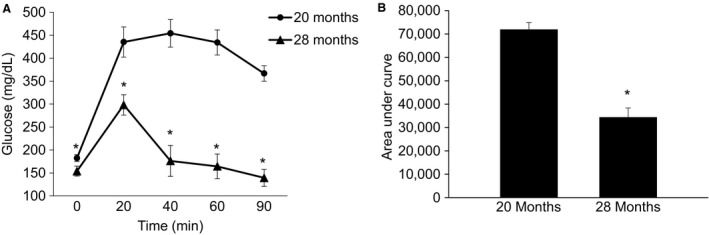
Aging from 20 to 28 months improves glucose tolerance in male C57BL/6J mice. (A) shows the blood glucose response to an intraperitoneal injection of glucose (2 g/kg). (B) is the area under the glucose tolerance curve. Following a significant ANOVA the LSD post hoc test was used to locate statistically significant differences. *Significantly different from 20‐month‐old mice by LSD post hoc test. *N* = 7 mice.

To further interrogate the idea that aging beyond 18–20 months improves glucose tolerance and reduces adiposity, we conducted GTT and assessed body composition 8 months following the completion of the MitoQ study. We clearly demonstrate that glucose tolerance improves markedly from 20 months of age to 28 months of age (Fig. 5). Similar to glucose tolerance, when body composition was followed in these same mice from 20 to 28 months of age we observed a significant decline in fat mass and percent body fat (Table 3).

**Table 3:** Body composition of 20 month and 28‐month‐old male C57BL/6J mice

	20 months (*n* = 7)	28 months (*n* = 7)	ANOVA *P*‐value
Body mass (g)	37.2 ± 1.9	33.6 ± 1.0	0.111
Fat mass (g)	12.5 ± 1.9	4.8 ± 0.9	0.015
Lean mass (g)	20.1 ± 0.3	23.4 ± 0.4	0.0001
% Body fat	32.5 ± 3.5	14.0 ± 2.2	0.007
Free body fluid (g)	3.0 ± 0.2	3.1 ± 0.2	0.814

## Discussion

This study clearly demonstrates that female C57BL/6J mice are protected from age‐related obesity and insulin resistance. Unlike females, male C57BL/6J mice are obese and severely insulin resistant by 18 months of age, a process that is completely reversed by 28 months. Unlike diet‐induced obesity, age‐related obesity is not improved by the mitochondrial targeted antioxidant MitoQ. Collectively, these findings are critical for contextualizing past and future work using the murine model to study potential sex differences in aging and diet‐induced obesity.

Recent epidemiological data indicates that obesity is greater in older individuals and that females have greater obesity prevalence than males (Ogden et al. 2014; Flegal et al. 2016). The effect of advancing age on obesity in preclinical mouse models is not well‐established. Our study indicates that male C57BL/6J mice gain substantially more fat mass with aging than female mice. Although estrogen can protect female mice from diet‐induced obesity (Stubbins et al. 2012), our 18‐month‐old female mice are well beyond the known onset of menopause in mice (8 months) and thus are deficient in estrogen (Diaz 2012). Since obesity results from an imbalance in energy expenditure and caloric intake, it would be expected that the AG male mice had lower energy expenditure and/or greater caloric intake than AG female mice and younger mice. This is not the case as no differences existed in energy expenditure between AG male and female mice, although AG female mice tended to have higher levels of total physical activity than aged males. Furthermore, caloric intake did not explain the large differences in adiposity between AG male and female mice. Perhaps the thermic effect of food, a variable we did not measure, may explain a portion of the differences in adiposity between AG male and female mice. To the best of our knowledge, there are no reports of sex differences in the thermic effect of food in mice or humans, and warrants further study.

MitoQ, a mitochondrial targeted antioxidant has been show to prevent diet‐induced obesity and insulin resistance (Fink et al. 2014). Our data shows that MitoQ, in the present dosing scheme, was ineffective at reducing adiposity and improving glucose tolerance in AG male C57BL/6J mice, suggesting that age‐related obesity and insulin resistance do not respond to this antioxidant treatment. However, although MitoQ can prevent diet induced obesity and insulin resistance, it seems to be less effective at reversing preexisting obesity due to a high fat diet (Fink et al. 2017). Because the ability of MitoQ to prevent weight gain from a high fat diet is due to changes in hypothalamic signaling and reduced food intake (Fink et al. 2014), perhaps AG mice are resistant to MitoQ's central effect, as we showed no changes in caloric intake in mice treated with MitoQ. Alternatively, due to its effect on water intake, we were forced to use a slightly lower dose of MitoQ than previous studies (Gioscia‐Ryan et al. 2014), a complicating factor that may have reduced the effectiveness of our intervention.

Our MitoQ study also failed to show the presence of age‐related insulin resistance, a finding that was clearly present approximately 2 months prior to the initiation of MitoQ treatment. Therefore, we tested the hypothesis that glucose tolerance improves as mice age from 20 to 28 months. In a repeated measures design, we observed a dramatic improvement in glucose tolerance as mice aged from 20 to 28 months, measured in the same animals (Fig. 5). Similar to the improvement in glucose tolerance, body composition was also improved from 20 to 28 months old. Although body mass did not decline significantly, fat mass and % body fat decreased dramatically from 20 to 28 months.

The mechanisms responsible for the observed reversal of age‐related obesity and insulin resistance are presently not known. Reductions in body mass are typically related to changes in energy homeostasis, with caloric expenditure exceeding caloric intake. Unfortunately, we did not assess food intake or energy expenditure as mice aged from 20 to 28 months as this age‐related reversal of obesity and insulin resistance was serendipitous and discovered retrospectively. Perhaps the improvement in glucose tolerance was due to enhanced insulin secretion (Leiter et al. 1988; Gregg et al. 2016; Krishna et al. 2016). Oh et al. (Oh et al. 2016) discovered that calcium sensing receptor (CaSR) expression in pancreatic *β*‐cells was substantially higher in 28‐month‐old C57BL/6J mice compared to 20‐month‐old mice without a change in plasma calcium levels. Since CaSR is involved with insulin secretion (Jones et al. 2007), an increase in insulin secretion from 20 to 28 months of age might have contributed to the improvement in glucose tolerance observed in the present study. However, with regards to obesity, it appears that increased adipocyte expression of CaSR would promote adiposity rather than decreasing it (He et al. 2011).

In summary, the present study demonstrates that female C57BL/6J mice are less susceptible to age‐related obesity and insulin resistance then male C57BL/6J mice. In an attempt to reduce the obesity and insulin resistance, we treated aged male mice with the mitochondrial‐targeted antioxidant, MitoQ. Unlike preexisting diet‐induced obesity, our findings indicate that age‐related obesity is not lessened by MitoQ treatment. Interestingly, we also show that the obesity and insulin resistant that was present in 18‐month‐old male mice is absent when the mice were again studied at 28 months of age, indicating that advancing age reduces adiposity and improves insulin sensitivity. These observations provide new insight into how aging impacts insulin action and body composition in male C57BL/6J mice, a mouse strain that has been utilized in thousands of studies examining metabolism and aging.

## Conflict of Interest

The authors have no conflicts of interest, financial or otherwise, to report.
